# The Characterization of R2R3-MYB Genes in Water Lily *Nymphaea colorata* Reveals the Involvement of NcMYB25 in Regulating Anthocyanin Synthesis

**DOI:** 10.3390/plants13212990

**Published:** 2024-10-26

**Authors:** Qi Liu, Shujuan Li, Tuanjie Li, Qian Wei, Yan Zhang

**Affiliations:** 1 Xi’an Botanical Garden of Shaanxi Province, Institute of Botany of Shaanxi Province, Shaanxi Engineering Research Centre for Conservation and Utilization of Botanical Resources, Xi’an 710061, China; bio_67@163.com (Q.L.);; 2 College of Life and Environmental Sciences, Minzu University of China, Beijing 100081, China; 3 Longcaoping Forestry Bureau of Shaanxi Province, Hanzhong 723400, China

**Keywords:** *Nymphaea colorata*, R2R3-MYB transcription factors, anthocyanins

## Abstract

*Nymphaea colorata*, valued for its diverse flower colors and attractive shapes, is a popular ornamental aquatic plant. Anthocyanins provide color to flowers, and their biosynthesis is regulated by the R2R3-MYB transcription factor. In this study, we identified and analyzed the R2R3-MYB genes in *N. colorata*, focusing on their structure, evolution, expression patterns, regulatory mechanisms, and biological functions. We also investigated the role of the *NcMYB25* gene in anthocyanin biosynthesis. There were 59 R2R3-MYB genes in *N. colorata*, distributed across 14 chromosomes. Among these, 14 genes were involved in segmental duplications and 6 in tandem duplications. Multiple R2R3-MYB transcription factors appeared to play a role in biological processes in *N. colorata*, including NcMYB48 in flavonoid synthesis, NcMYB33 in lignin synthesis, NcMYB23 in cold stress response, and NcMYB54 in osmotic stress response. Additionally, we identified 92 miRNAs in *N. colorata*, with 43 interacting with 35 R2R3-MYB genes. The NcMYB25 protein is localized in the nucleus and possesses transcriptional activation activity. Overexpression of the *NcMYB25* gene in an apple pericarp resulted in anthocyanin accumulation. These findings provide insight into the evolutionary trajectory of the R2R3-MYB genes in *N. colorata* and highlight the regulatory function of the *NcMYB25* gene in anthocyanin biosynthesis.

## 1. Introduction

The term “water lily” refers to plants in the genus *Nymphaea* within the family Nymphaeaceae. Ecologically, water lilies are divided into tropical and hardy varieties, with the former mainly found in tropical regions and the latter found in subtropical and temperate regions [1]. Currently, there are over 50 species and 1000 horticultural cultivars of water lilies [2,3]. Due to their diverse flower colors, aroma, and shapes, water lilies are often used in garden water features, indoor floral decorations, and floral arrangements. Water lilies have extensive root systems and require substantial amounts of nitrogen and phosphorus during growth. They also have a strong ability to absorb heavy metals like mercury, chromium, and manganese from water, making them valuable for aquatic ecological restoration [4]. Moreover, as one of the basal angiosperms, water lilies are important for studying early plant evolution [5]. *Nymphaea colorata* is renowned for its blue flowers. As a significant ornamental aquatic plant, breeding new varieties with different flower colors is a current focus in cultivating water lily varieties. The high-quality genome of *N. colorata* has recently been published, greatly facilitating the analysis of evolution, gene functions, and molecular regulatory networks in this species [6].

In plants, including *N. colorata*, flower coloration is often closely related to the types and content of anthocyanins in the petals. Anthocyanins are important components of plant flavonoids and are typically found in multiple plant tissues, giving them vibrant colors [7]. The color of flowers not only contributes to their ornamental value but plays a significant role in pollen dispersal and UV radiation tolerance [7,8]. Key enzymes in the anthocyanin biosynthesis pathway have been identified and analyzed in various plants. The synthesis of anthocyanins starts with the reaction between malonyl-CoA and coumaroyl-CoA catalyzed by chalcone synthase (CHS). Naringenin chalcone is then converted into anthocyanidins through the subsequent actions of chalcone isomerase (CHI), flavanone 3-hydroxylase (F3H), flavonoid 3′,5′-hydroxylase (F3′5′H), dihydroflavonol 4-reductase (DFR), and anthocyanin synthase (ANS) [9,10]. Finally, anthocyanidins are converted into stable and soluble anthocyanin through the action of the flavonoid-3-O-glucosyltransferase (UFGT) [11,12]. Genes affecting anthocyanin synthesis in plants are divided into two categories. One category includes the genes involved directly in the anthocyanin biosynthesis pathway, such as DFR and ANS. Overexpression of the *HvDFR* gene from *Hosta ventricosa* increased the accumulation of anthocyanins in transgenic tobacco [13], and overexpression of the *TaDFR-1* gene in wheat led to a significant accumulation of anthocyanin in transgenic *Arabidopsis* [14]. The other category includes the transcription factors that regulate the expression of key genes in the anthocyanin biosynthesis pathway, including R2R3-MYB transcription factors. These factors regulate anthocyanin synthesis by activating or inhibiting the transcription of genes related to the anthocyanin biosynthesis pathway [15].

MYB proteins are among the largest transcription factor families in plants and are involved in various biological processes [16]. MYB proteins are characterized by a highly conserved DNA-binding domain known as the MYB domain, which typically consists of no more than four repeats (R) of about 52 amino acids each, forming three alpha-helices. The second and third helices of each R form a helix-turn-helix (HTH) structure with three regularly spaced tryptophan (or hydrophobic) residues, creating a hydrophobic core in the 3D HTH structure [17]. MYB proteins are categorized into different classes based on the number and type of adjacent R: 1R-MYB, R2R3-MYB, 3R-MYB, and 4R-MYB. Among these, R2R3-MYB proteins are the most numerous and involved in the widest range of biological processes, including development, metabolism, and responses to biotic and abiotic stresses [18]. One hypothesis is that R2R3-MYB genes evolved from ancestral R1R2R3-MYB genes by losing the R1 sequence during evolution, leading to the formation of the R2R3-MYB gene family [18,19]. Many R2R3-MYB transcription factors have been characterized, participating in the regulation of primary and secondary metabolism, growth, development, and responses to biotic and abiotic stresses. Studies have shown that several R2R3-MYB transcription factors regulate flavonoid biosynthesis, such as AtMYB11, AtMYB12, and AtMYB111, which are involved in the synthesis of flavonols [20], and AtMYB75, AtMYB90, AtMYB113, and AtMYB114, which regulate anthocyanin synthesis [21].

Flower color is an eye-catching phenotype and a significant source of both ornamental and commercial value for plants [22]. The biosynthesis of anthocyanins, the basis of flower coloration, is typically regulated by multiple R2R3-MYB transcription factors. Some transcription factors regulating anthocyanin synthesis have been identified. In *Arabidopsis*, AtMYB75, AtMYB90, AtMYB113, and AtMYB114 act as positive regulators in the anthocyanin biosynthesis pathway [21,23]. Transient expression of the *MdMYB10* gene enhanced the accumulation of anthocyanin in transgenic tobacco and apples [15]. In a mature pear pericarp, the expression level of *PyMYB10* gene was positively correlated with *CHS*, CHI, *DFR*, and *ANS* genes [24]. Overexpression of the *MdMYB3* gene in tobacco activated the transcription of *CHS*, *CHI*, and *UFGT* genes, leading to the accumulation of anthocyanin in flowers [25]. Furthermore, some studies have shown that R2R3-MYB transcription factors influence the accumulation of anthocyanins by binding to the promoters of target genes and regulating their expression. The LvMYB1 transcription factor activated the transcription of the *LvANS* gene by binding to its promoter, thereby promoting the accumulation of anthocyanins [26]. With the publication of the high-quality genome of *N. colorata*, some key enzymes in the anthocyanin biosynthesis pathway have been identified. However, the R2R3-MYB transcription factor, an important regulator in anthocyanin synthesis, its quantity, types, and how it affects anthocyanin synthesis remains unclear.

In this study, we first identified the R2R3-MYB gene family members in *N. colorata* based on high-quality genomic data and then analyzed their structures, evolution, expression patterns, and regulatory networks. Finally, we focused on the biological functions of the NcMYB25 transcription factor in anthocyanin biosynthesis and explored the molecular mechanisms underlying flower color formation in *N. colorata*.

## 2. Results

### 2.1. Identification and Physicochemical Properties of R2R3-MYB Proteins in N. colorata

**Figure 1:**
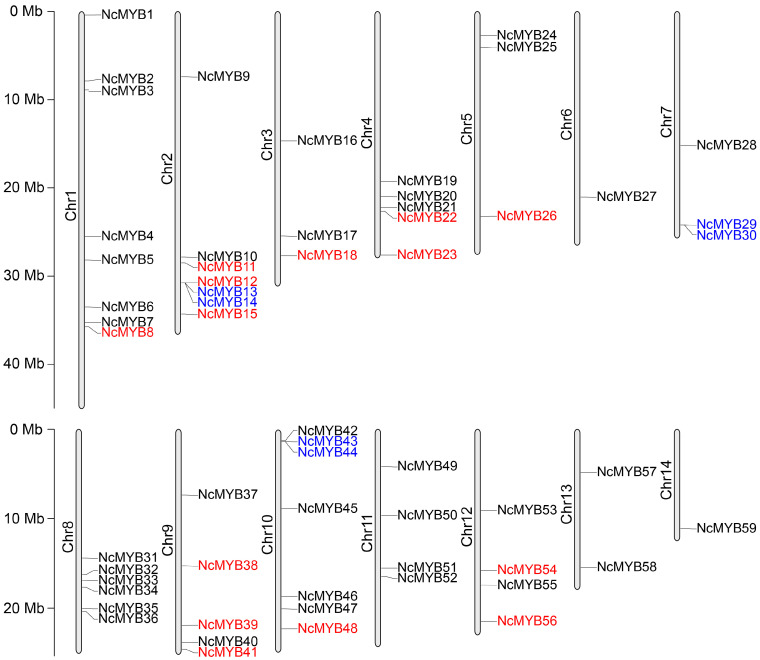
Distribution of R2R3-MYB genes on the chromosomes in *N. colorata*. The scale on the left side of the image represented the length of the chromosomes. Genes labeled in red indicate segmental duplication genes, while genes labeled in blue indicate tandem duplication genes.

A total of 59 R2R3-MYB genes were identified in the *N. colorata* genome, distributed across 14 chromosomes. Chromosome 1 harbored the highest number, with 8 genes, followed by chromosomes 2 and 10 with 7 genes each, while chromosomes 6 and 14 contained only 1 gene each (Figure 1). The amino acid number of *N. colorata* R2R3-MYB proteins ranged from 142 to 513, with molecular weights from 16.39 to 55.06 kDa. The NcMYB4 protein possessed the largest molecular weight, and the NcMYB21 protein possessed the smallest. Based on theoretical isoelectric points (pI), the R2R3-MYB proteins were categorized into acidic (32 proteins) and basic (27 proteins). All R2R3-MYB proteins in *N. colorata* were hydrophilic (Table S1).

### 2.2. Structural Analysis of R2R3-MYB in N. colorata

**Figure 2:**
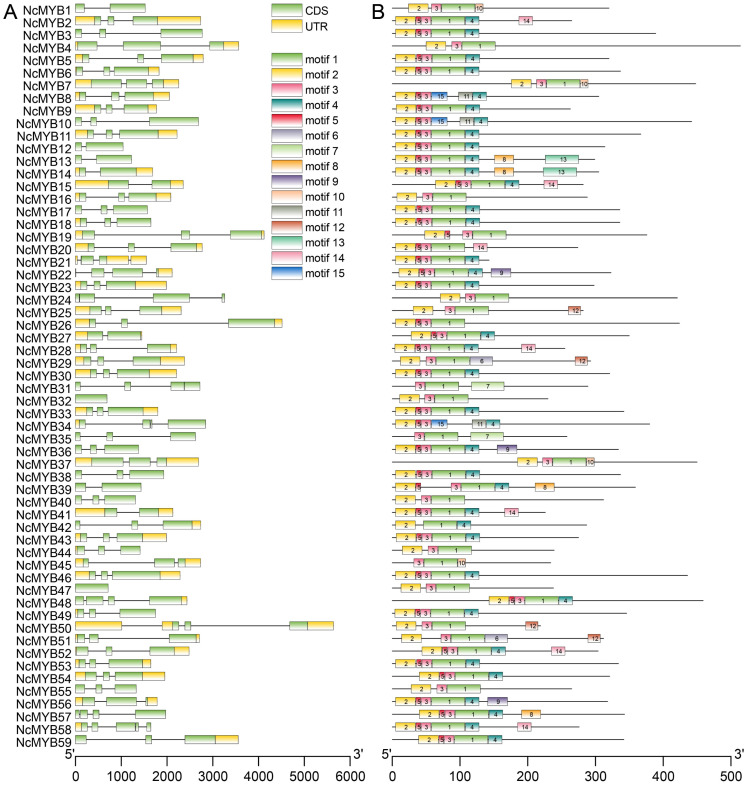
Structural analysis of R2R3-MYB in *N. colorata*. (**A**) Gene structure of R2R3-MYB genes. The scale bar at the bottom represented gene length. Yellow rectangles indicate untranslated regions (UTR), while green rectangles represent coding sequence (CDS). (**B**) The conserved motif of R2R3-MYB transcription factors. The scale bar at the bottom represents the number of amino acids. Each rectangle represents a motif, with detailed sequences provided in Figure S1.

To analyze the structure of the R2R3-MYB gene, exon–intron analysis was performed. The R2R3-MYB genes in *N. colorata* showed generally fewer introns, with *NcMYB58* gene possessing the most at four introns. Forty R2R3-MYB genes contained two introns, and *NcMYB32* and *NcMYB47* genes possessed no intron (Figure 2A). To analyze the protein structure of R2R3-MYB in *N. colorata*, a MEME analysis was performed. Motif2, motif3, and motif5 were located within the R2 domain, while motif1 and motif4 were located in the R3 domain. Motif3 was present in all R2R3-MYB proteins in *N. colorata*, motif1 in 56 members, motif2 in 54, motif4 in 36, and motif5 in 39. Other motifs were found in only a few proteins (Figure 2B and Figure S1).

### 2.3. Multiple Sequence Alignment and Phylogenetic Analysis

**Figure 3:**
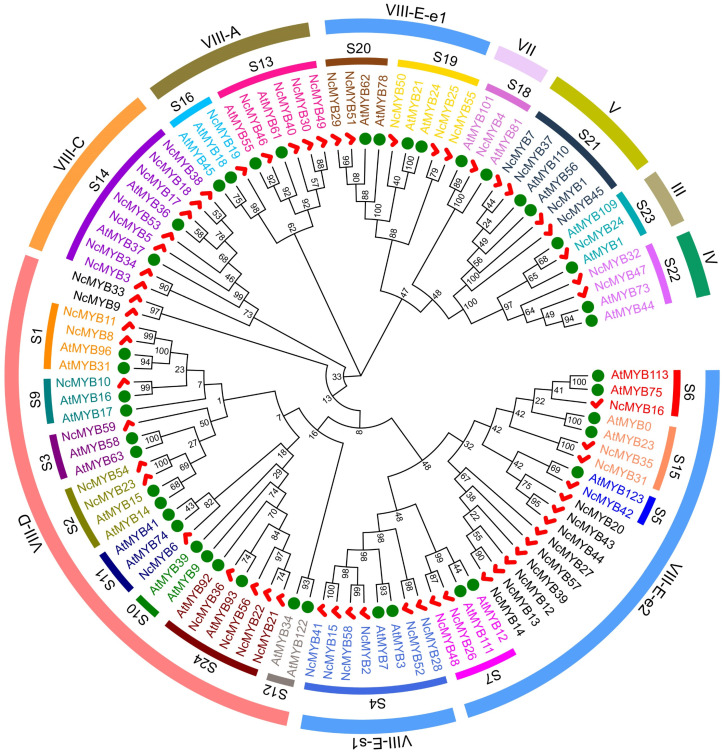
Phylogenetic analysis of R2R3-MYB proteins in *N. colorata*. The R2R3-MYB proteins of *N. colorata* were classified based on two classification schemes used for R2R3-MYB proteins in *Arabidopsis*. Green circles represent R2R3-MYB proteins in *Arabidopsis*, while red checks represent R2R3-MYB proteins in *N. colorata*.

To investigate the phylogeny of R2R3-MYB proteins in *N. colorata*, multiple sequence alignments and a phylogenetic tree were constructed. The conserved R2 and R3 domains were present in all proteins in *N. colorata* (Figure S2). A phylogenetic tree was constructed using all R2R3-MYB protein sequences in *N. colorata* and partial R2R3-MYB protein sequences in *Arabidopsis*. The majority of R2R3-MYB proteins in *N. colorata* were correctly classified, with the highest numbers in groups S4 and S14 (Figure 3). There were no members in groups S10 and S12 in *N. colorata*. Using an alternative classification rule, the R2R3-MYB proteins in *N. colorata* were divided into 10 groups, with the most members in groups VIII-D and VIII-E-e2 and the fewest in group VII. Proteins in groups VIII-E-e1, III, IV, V, and VII clustered together, suggesting closer evolutionary relationships and potential functional similarities (Figure 3).

### 2.4. Evolutionary Analysis of R2R3-MYB Genes in N. colorata

**Figure 4:**
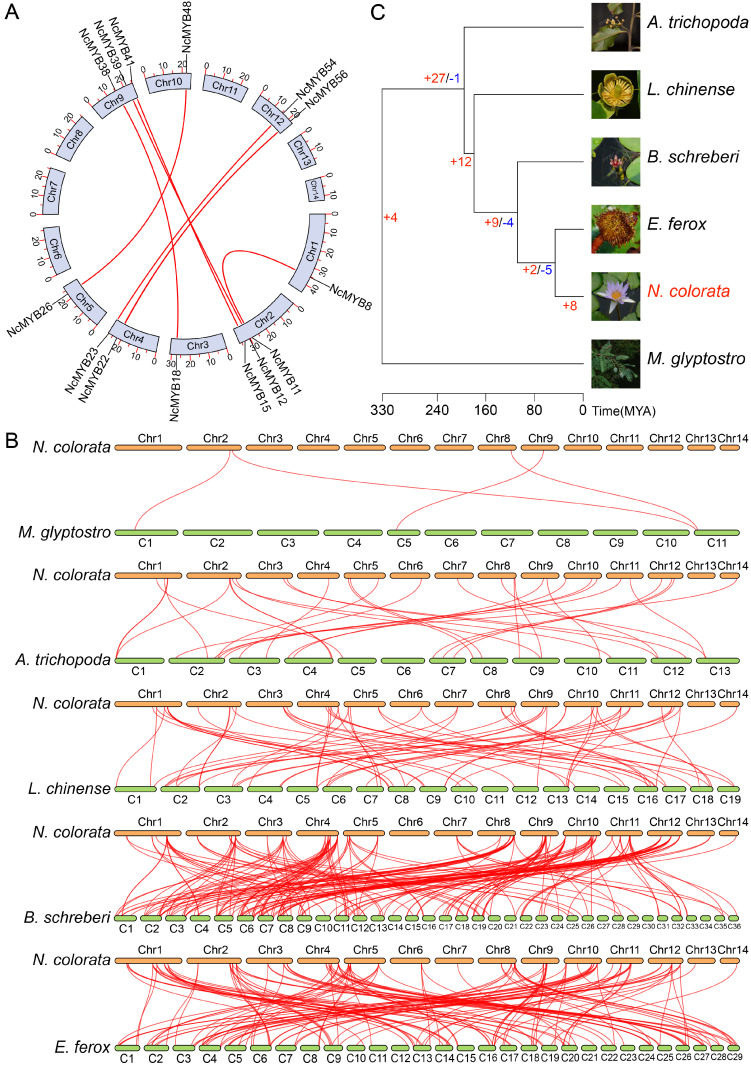
Evolutionary analysis of R2R3-MYB genes in *N. colorata*. (**A**) Segmental duplication genes of R2R3-MYB genes in *N. colorata*. (**B**) Collinearity analysis between *N. colorata* and five other species. (**C**) Evolutionary history of R2R3-MYB genes in *N. colorata*. Species trees and divergence times between species were obtained from the TimeTree website. Red-labeled numbers indicate newly evolved genes, while blue numbers indicate lost genes. Species illustrations were from the Plant Photo Bank of China (PPBC).

To analyze the evolution of the R2R3-MYB gene family, a collinearity analysis was performed. The collinearity analysis revealed that 14 genes underwent segmental duplication and 6 genes underwent tandem duplication (Figure 1 and Figure 4A). The gene duplication event between *NcMYB8* and *NcMYB11* genes likely derived from a recent whole genome duplication (WGD) event, while duplications between *NcMYB23* and *NcMYB54* genes likely occurred during an earlier WGD event (Figure S3 and Table S2).

To analyze the evolutionary history of the R2R3-MYB gene family in *N. colorata*, a collinearity analysis was executed between *N. colorata* and *M. glyptostroboides*, *A. trichopoda*, *L. chinense*, *B. schreberi*, and *E. ferox*, respectively. The number of homolog genes was correlated with the phylogenetic relationships and the chromosome numbers of the genomes analyzed (Figure 4B,C).

### 2.5. Cis-Acting Elements Analysis of R2R3-MYB Genes in N. colorata

**Figure 5:**
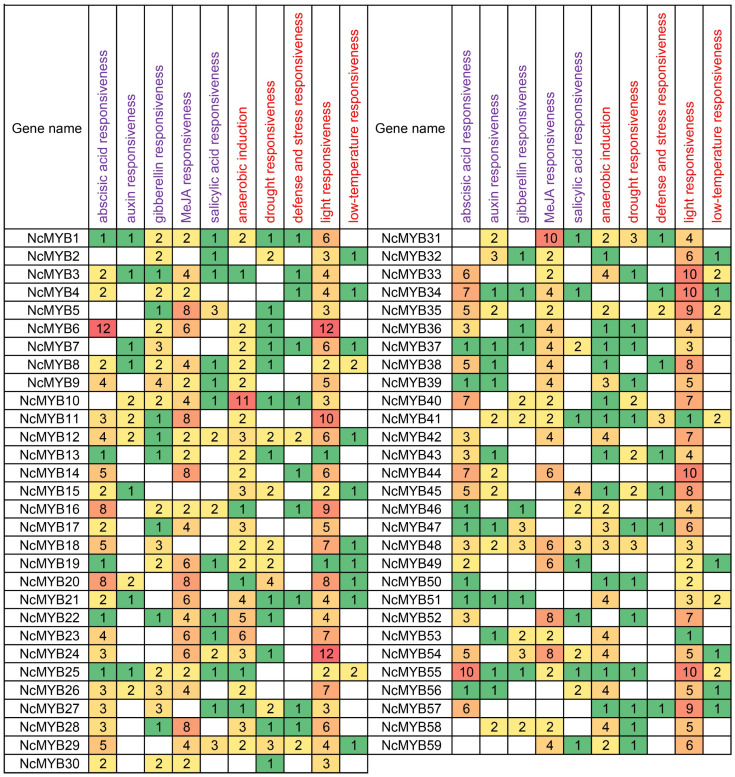
The *cis*-acting elements of the R2R3-MYB gene promoter. Purple labels indicate hormone-related *cis*-acting elements, red labels denote environmental stress-related *cis*-acting elements, and numbers represent the quantity of *cis*-acting elements. The colored boxes in the figure represent the number of *cis*-acting elements, with the gradient from green to orange indicating an increasing number.

To explore their potential biological functions, the *cis*-acting elements in R2R3-MYB gene promoters were analyzed. The promoters of R2R3-MYB genes in *N. colorata* contained the *cis*-acting elements responsive to hormone signals and environmental stress. ABA-responsive *cis*-acting elements were found in 49 R2R3-MYB gene promoters, auxin-responsive *cis*-acting elements in 30, gibberellin-responsive *cis*-acting elements in 37, MeJA-responsive *cis*-acting elements in 46, and SA-responsive *cis*-acting elements in 29 (Figure 5). Most promoters of R2R3-MYB genes in *N. colorata*, except those of *NcMYB50* and *NcMYB57* genes, possessed more than one type of hormone-responsive element. Light-responsive *cis*-acting elements were present in all promoters. Additionally, 37 gene promoters possessed drought stress-responsive elements, and 23 genes possessed cold stress-responsive elements. Fourteen gene promoters possessed *cis*-acting elements responsive to both cold and drought stress, indicating possible involvement in the responses to drought and cold stress (Figure 5).

### 2.6. Expression Patterns Analysis of R2R3-MYB Genes in N. colorata

**Figure 6:**
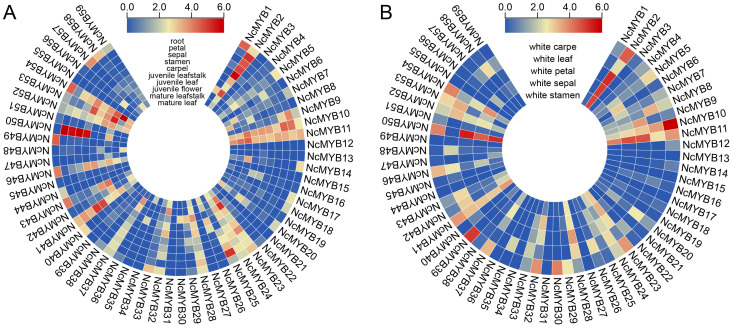
Expression pattern of R2R3-MYB genes in different tissues based on transcriptome data. (**A**) Expression patterns of R2R3-MYB genes in *N. colorata* petals. (**B**) Expression patterns of R2R3-MYB genes in *White colorata* petals.

An expression analysis revealed significant variation in R2R3-MYB genes in *N. colorata* across different tissues. Notably, several genes exhibited tissue-specific expression, suggesting specialized functions. *NcMYB50* and *NcMYB54* genes showed high expression in stamens, sepals, and petals, indicating a role in flower organ development. *NcMYB10* and *NcMYB41* genes were expressed in multiple tissues, with higher expression in reproductive organs and leaves, suggesting roles in both vegetative and reproductive growth. *NcMYB17* and *NcMYB57* genes exhibited strong root-specific expression, indicating roles in root biology (Figure 6A). In *Nymphaea White colorata*, the *NcMYB11* gene was highly expressed in sepals and petals. The *NcMYB50* gene was highly expressed in sepals, petals, and carpels, especially in petals, suggesting its role in flower development. *NcMYB10*, *NcMYB39*, and *NcMYB54* genes were notably expressed in carpels, implying specific roles in carpel development or function. *NcMYB2*, *NcMYB23*, and *NcMYB41* genes were expressed in multiple tissues, indicating involvement in various physiological functions (Figure 6B).

**Figure 7:**
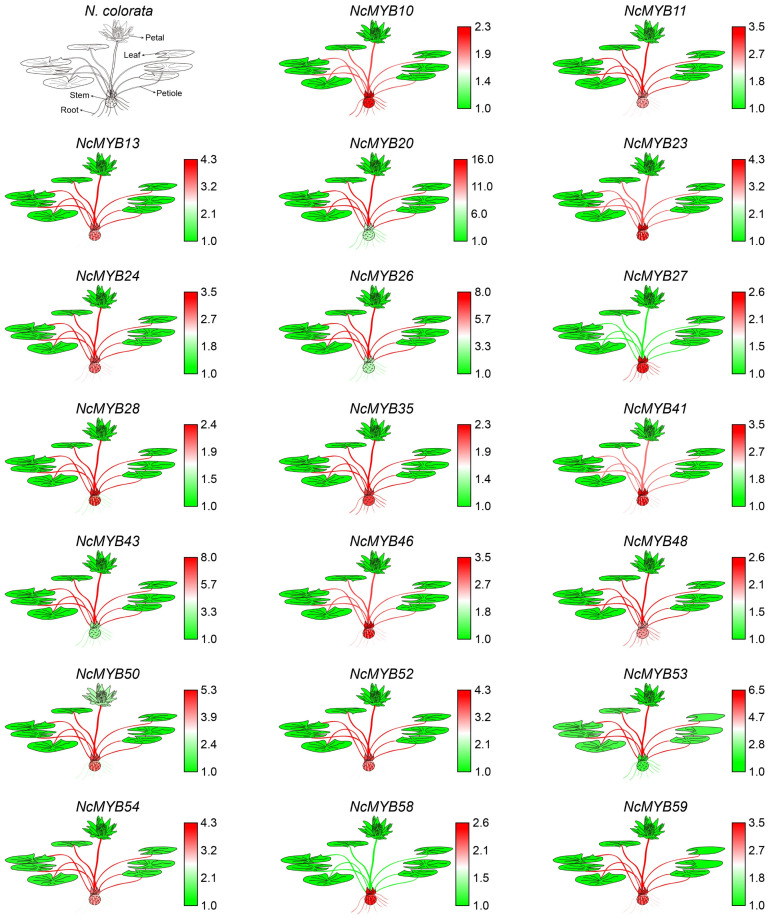
Expression levels of R2R3-MYB genes in *N. colorata* based on qRT-PCR analysis.

To further validate the expression patterns, a qRT-PCR analysis of R2R3-MYB genes in roots, stems, leaves, petioles, and petals was performed. The qRT-PCR results showed higher expression levels of most R2R3-MYB genes in petioles and stems, and lower expression in leaves and petals. In petiole tissues, *NcMYB26* and *NcMYB43* genes were highly expressed, while *NcMYB2* and *NcMYB58* genes were low. In stems and petals, *NcMYB50* and *NcMYB53* genes showed the highest expression levels, with *NcMYB53* genes highest in leaves (Figure 7).

### 2.7. PPI Analysis of R2R3-MYB Proteins in N. colorata

**Figure 8:**
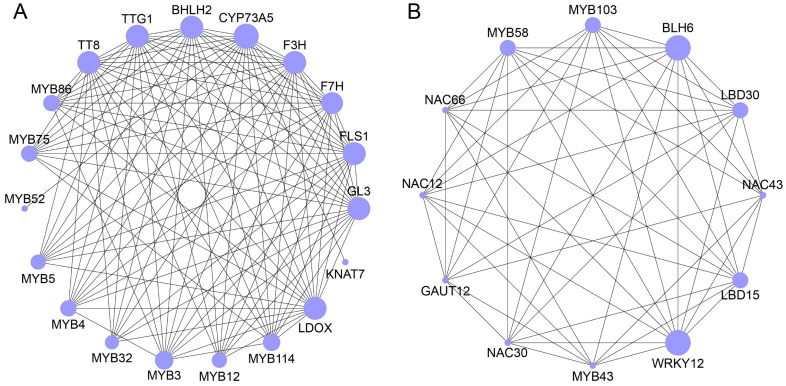
Core network of PPI analysis for R2R3-MYB in *N. colorata*. (**A**) Cluster 1 module. (**B**) Cluster 2 module.

To elucidate the complex biological processes involving R2R3-MYB proteins in *N. colorata*, a PPI analysis was conducted. The PPI network included 56 nodes and 374 edges, involving various complex biological processes (Figure S4). Two core modules, Cluster 1 and Cluster 2, were identified using the MCODE plugin in Cytoscape v3.8.2 software. Cluster 1, with 19 nodes and 113 edges, was associated with flavonoid biosynthesis (Figure 8A). MYB114, TT8, MYB75, and LDOX regulated the synthesis of flavonoids and anthocyanins, while F3H and FLS were key enzymes in the flavonoid biosynthesis pathway. Additionally, the MYB32, MYB52, and MYB86 transcription factors regulated flavonoid synthesis (Figure 8A). Cluster 2, with 12 nodes and 52 edges, was related to plant growth, secondary metabolism, and stress responses (Figure 8B). MYB103, MYB43, and MYB58 participate in the regulation of cell wall components and lignin synthesis, while LBD15, LBD30, and BLH6 are involved in root, shoot, and flower organ formation. NAC30, NAC43, and NAC66 are associated with responses to drought and cold stress (Figure 8B). These findings suggest that R2R3-MYB proteins in *N. colorata* might regulate flavonoid biosynthesis, development regulation, and stress response.

### 2.8. GO Functional Analysis of R2R3-MYB Proteins in N. colorata

**Figure 9:**
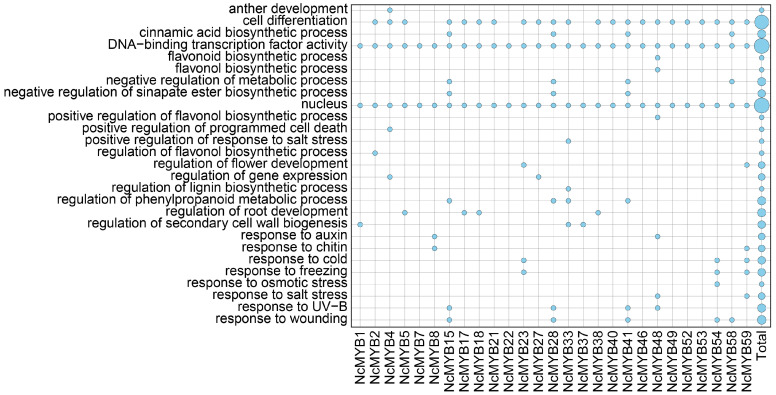
GO functional analysis of R2R3-MYB proteins in *N. colorata*. The *x*-axis represents R2R3-MYB proteins, while the *y*-axis represents GO terms. Larger circles indicate a greater number of proteins.

To analyze the functions of R2R3-MYB proteins in *N. colorata*, a GO functional annotation analysis was performed. A total of 27 R2R3-MYB proteins in *N. colorata* were annotated in the GO database, encompassing multiple terms across cellular component, biological process, and molecular function categories (Figure 9 and Table S3). All R2R3-MYB proteins in *N. colorata* are located in the nucleus and possess DNA-binding activity. The R2R3-MYB proteins in *N. colorata* likely participate in various biological processes, including stress responses, growth regulation, and secondary metabolism. *NcMYB23*, *NcMYB48*, *NcMYB54*, and *NcMYB59* genes may respond to cold, freezing, osmotic, and salt stresses. In growth regulation, four genes affect root growth, and three genes influence flower development (Figure 9). For secondary metabolism, NcMYB48 regulate flavonoid synthesis, NcMYB33 regulate lignin synthesis, and NcMYB15, NcMYB18, NcMYB41, and NcMYB58 are involved in cinnamic acid synthesis (Figure 9).

### 2.9. Regulation of R2R3-MYB Gene Expression by miRNAs in N. colorata

**Figure 10:**
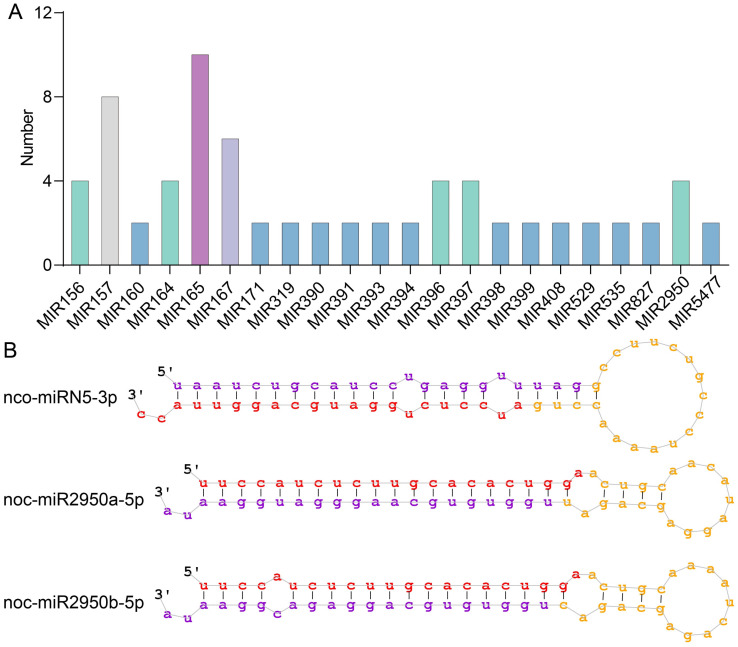
Identification of miRNAs in *N. colorata*. (**A**) Statistics of identified miRNA family members. (**B**) Representative stem–loop structures of identified miRNA precursors. Red is the mature miRNA sequence, purple is the miRNA-star sequence, and yellow is the non-miRNA and loop sequences in the stem.

To investigate the regulatory mechanisms of the R2R3-MYB gene in *N. colorata*, miRNAs were identified in *N. colorata*, and their regulatory relationships with R2R3-MYB genes were analyzed. A total of 92 miRNAs were identified in *N. colorata*, including 72 conserved miRNAs and 20 non-conserved miRNAs (Figure 10A and Table S4). All conserved miRNAs belong to 22 families, with the highest number of members in the MIR165, MIR157, and MIR167 families, possessing 10, 8, and 6 members, respectively (Figure 10A and Table S4). The precursor sequences of all miRNAs exhibit typical stem–loop structure (Figure 10B).

**Figure 11:**
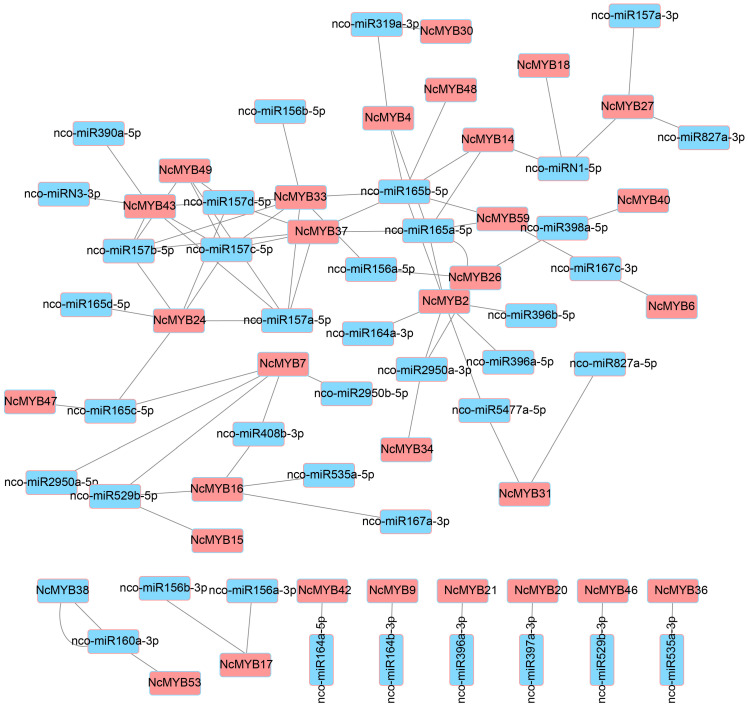
Regulation of R2R3-MYB gene expression miRNA in *N. colorata*. The blue-filled rectangle with a red border represents the miRNA in *N. colorata*, and the red-filled rectangle with a blue border represents the R2R3-MYB gene in *N. colorata*. Gray lines indicate potential regulatory relationships between the miRNAs and R2R3-MYB genes.

To investigate the regulation of R2R3-MYB gene expression by miRNAs, the potential miRNAs that regulate R2R3-MYB gene expression were predicted. A total of 84 regulatory relationships were found between 43 miRNAs and 35 R2R3-MYB genes. Specifically, 20 R2R3-MYB genes may be regulated by a single miRNA each, two R2R3-MYB genes could be regulated by two miRNAs, and 13 R2R3-MYB genes could be potentially regulated by at least three miRNAs. Notably, both *NcMYB2* and *NcMYB33* genes could be regulated by seven different miRNAs. Among these miRNAs, 26 targeted a single R2R3-MYB gene each, 7 targeted two genes, and nco-miR165b-5p targeted the most genes, with a total of 7 (Figure 11).

### 2.10. NcMYB25 May Be Involved in the Regulation of Anthocyanin Synthesis in N. colorata

**Figure 12:**
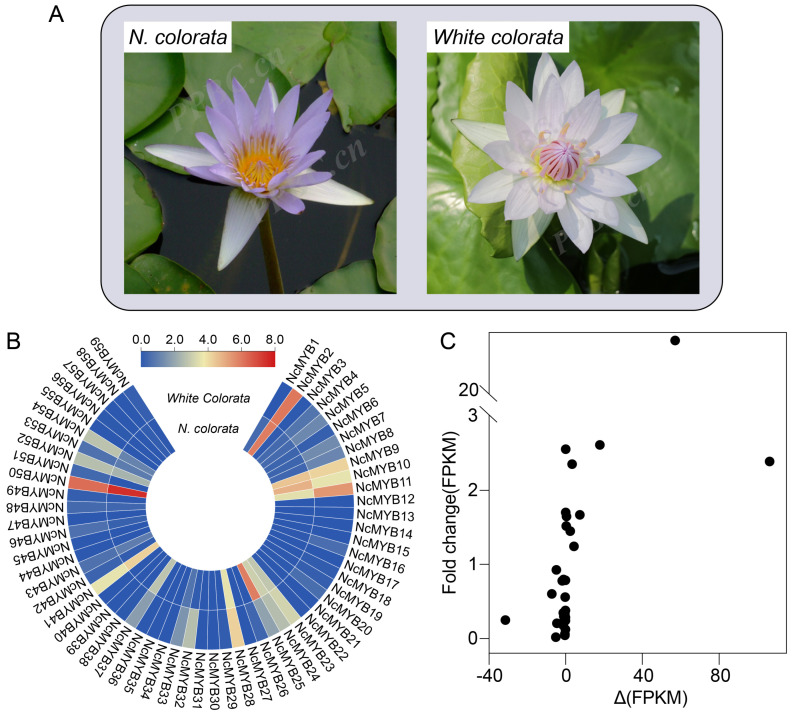
The expression levels of the R2R3-MYB gene in *N. colorata* and *White colorata* in petals. (**A**) Representative images of *N. colorata* and *White colorata*. Images were from PCBB. (**B**) Expression levels of R2R3-MYB genes in *N. colorata* and *White colorata* in petals. (**C**) Scatter plot of the expression levels of R2R3-MYB gene. The expression level of the MYB genes in *N. colorata* was denoted as FPKM (*N. colorata*). Similarly, the expression level of the MYB genes in *White colorata* was denoted as FPKM (*White colorata*). The *x*-axis shows the difference in the expression level of the R2R3-MYB gene between *N. colorata* and *White colorata*, and the difference was calculated as ΔFPKM = FPKM (*N. colorata*) – FPKM (*White colorata*). The *y*-axis shows the fold change of the expression level of R2R3-MYB gene between *N. colorata* and *White colorata*. The fold change was calculated using the formula: Fold Change (FPKM) = FPKM (*N. colorata*)/FPKM (*White colorata*).

To identify the R2R3-MYB transcription factors potentially regulating anthocyanin synthesis in *N. colorata*, the expression levels of R2R3-MYB genes in the petals of *N. colorata* and *White colorata* were compared (Figure 12A,B). Compared to *White colorata*, 12 R2R3-MYB genes exhibited higher expression levels in *N. colorata*, with *NcMYB10*, *NcMYB25*, and *NcMYB50* showing the highest expression. Among the R2R3-MYB genes analyzed, five showed differential expression fold changes greater than 2 between *N. colorata* and *White colorata*. Notably, the *NcMYB25* gene exhibited the highest fold change, reaching 21.73 (Figure 12B). Based on gene expression levels and fold changes, the *NcMYB25* gene was selected for further analysis to investigate its role in anthocyanin synthesis.

### 2.11. Localization of NcMYB25 Protein in the Nucleus

**Figure 13:**
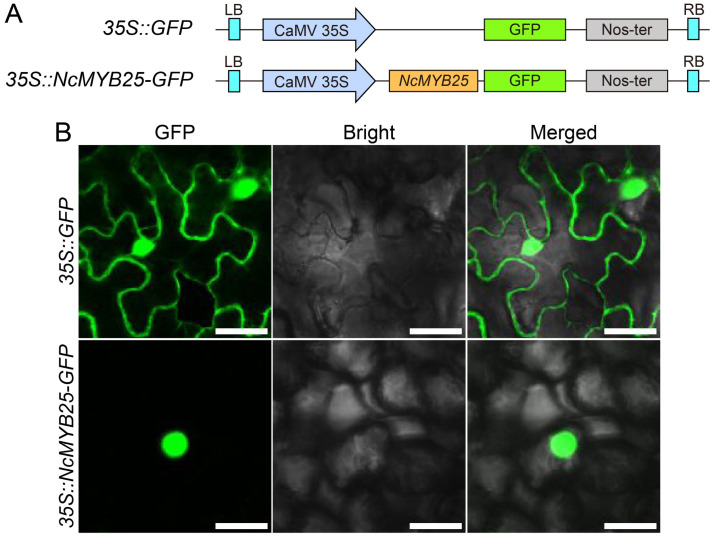
Subcellular localization analysis of the NcMYB25 protein. (**A**) Schematic diagrams of the *35S::GFP* and *35S::NcMYB25-GFP* vectors used for subcellular localization. (**B**) Fluorescence signals of the GFP protein and the NcMYB25-GFP fusion protein in tobacco leaf cells observed under a laser confocal microscope. Scale bar = 25 µm.

To verify whether the NcMYB25 protein was localized in the nucleus, a subcellular localization analysis was conducted using the tobacco expression system (Figure 13A). In tobacco leaves transformed with the *35S::GFP* vector, green fluorescence was observed around the cell nucleus and cell membrane. By contrast, tobacco leaves transformed with the *35S::NcMYB25-GFP* vector showed green fluorescence exclusively in the nuclear region, indicating that the NcMYB25 protein was localized in the nucleus (Figure 13B).

### 2.12. Transcriptional Activation Activity of NcMYB25 Transcription Factors

**Figure 14:**
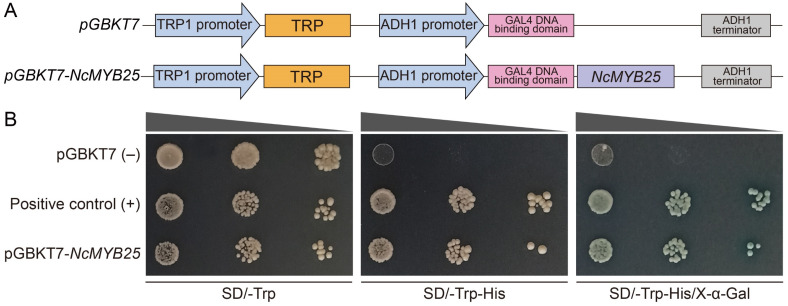
Transcriptional activation activity analysis of the NcMYB25 transcription factor. (**A**) Schematic diagrams of the *pGBKT7* and *pGBKT7-NcMYB25* vectors used in the transcriptional activation activity assay. (**B**) Representative images of the transcriptional activation activity analysis results, with pGBKT7 as the negative control.

To analyze the transcriptional activation activity of the NcMYB25 transcription factor, we conducted an assay using the *pGBKT7* vector and the AH109 yeast system (Figure 14A). Yeast cells expressing the *NcMYB25* gene and positive control yeast cells grew normally on media lacking tryptophan and media lacking both tryptophan and histidine, while negative control yeast cells only grew normally on media lacking tryptophan, indicating that the NcMYB25 transcription factor possesses transcriptional activation activity (Figure 14B).

### 2.13. Overexpression of NcMYB25 Gene Enhanced the Accumulation of Anthocyanins in Apple Pericarp

**Figure 15:**
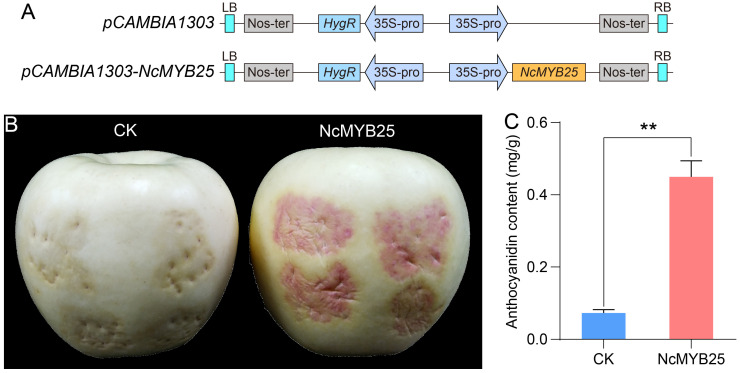
Overexpression of *NcMYB25* gene enhanced the accumulation of anthocyanins in an apple pericarp. (**A**) Schematic diagrams of the *pCAMBIA1303* and *pCAMBIA1303-NcMYB25* vectors used in the apple pericarp transient transformation assay. (**B**) The color change in the apple pericarp after *Agrobacterium* injection. (**C**) Anthocyanin content. “**” indicated significance at *p* < 0.01.

To study the effect of the NcMYB25 transcription factor on anthocyanin synthesis, the *NcMYB25* gene was transiently expressed in an apple pericarp, and anthocyanin accumulation was measured (Figure 15A). Compared to that of the control, the apple pericarp expressing the *NcMYB25* gene showed distinct red coloration (Figure 15B). Compared to the control, the anthocyanin content in the NcMYB25 group was significantly higher (Figure 15C). These results showed that overexpression of *NcMYB25* gene increased the accumulation of anthocyanin in the transgenic apple pericarp, suggesting that the NcMYB25 transcription factor played a positive regulatory role in anthocyanin synthesis.

## 3. Discussion

Water lily plays an increasingly important role in water gardens, water purification, and ecological civilization construction. *N. colorata* is a typical tropical water lily with striking flowers and high ornamental value [27]. Flower color has long been a focal point in water lily breeding and research. In the formation of anthocyanins, the fundamental compounds of flower color, R2R3-MYB transcription factors act as crucial regulatory factors [10,28]. Numerous R2R3-MYB transcription factors have been identified and analyzed across various species, revealing substantial variation in their numbers among different species. In the *Marchantia polymorpha* genome, there were 29 R2R3-MYB genes; in *Salvinia cucullata*, 41 genes; in *Gnetum montanum*, 37 genes; in *Oryza sativa*, 113 genes; in *Vitis vinifera*, 130 genes; and in *Glycine max*, 298 genes [16]. The differences in the number of R2R3-MYB genes among species relate to various factors, such as the evolutionary stage of the species, WGD events, and the quality of genome sequencing and annotation [16]. Generally, higher plants have more R2R3-MYB genes compared to lower plants, and species that have undergone more WGD events tend to have a higher number of R2R3-MYB genes. In this study, 59 R2R3-MYB genes were identified in the *N. colorata*. The water lily belongs to one of the early differentiation groups of angiosperms, and the relatively small number of R2R3-MYB genes in *N. colorata* may reflect its evolutionary status [6]. On the other hand, the relatively stringent criteria used in gene identification in this study may also contribute to the lower number of identified R2R3-MYB genes in *N. colorata*.

Based on phylogenetic and evolutionary analysis results, the evolutionary history of R2R3-MYB genes in *N. colorata* was inferred. *M. glyptostroboides*, as a representative of gymnosperms, contained four orthologous genes of the R2R3-MYB genes in *N. colorata*. It is speculated that *NcMYB11*, *NcMYB12*, *NcMYB36*, and *NcMYB39* genes may be the oldest members of the R2R3-MYB gene family in *N. colorata*. *A. trichopoda* is one of the earliest diverging angiosperms with no phylogenetic relationship to most other angiosperms. The genome of *A. trichopoda* contained 30 orthologous genes of the R2R3-MYB genes in *N. colorata*, suggesting that 27 orthologous genes of the R2R3-MYB genes in *N. colorata* had newly emerged during the evolution from gymnosperms to angiosperms, and the NcMYB25 gene may have formed during this historical stage. When the most recent common ancestor (MRCA) of *A. trichopoda* and *L. chinense* diverged into *L. chinense*, 12 orthologous genes of the R2R3-MYB genes in *N. colorata* had newly emerged. When the MRCA of *L. chinense* and *B. schreberi* diverged into *B. schreberi*, 9 orthologous genes of the R2R3-MYB genes in *N. colorata* had newly emerged. *B. schreberi*, as a unique group among aquatic plants, provides valuable insights into the origins, differentiation, and evolutionary history of aquatic plant groups. During the evolutionary stage when the MRCA of *B. schreberi* and *E. ferox* diverged into *E. ferox*, the orthologous genes of the *NcMYB44* and *NcMYB57* genes were newly formed. Additionally, when the MRCA of *E. ferox* and *N. colorata* diverged into *N. colorata*, eight R2R3-MYB genes in *N. colorata* were newly emerged (Table S5).

The classification of the R2R3-MYB transcription factor remains contentious, primarily due to lineage-specific gene loss or expansion [16,29,30]. Currently, the two classifications of R2R3-MYB proteins in *Arabidopsis* are often used as references for classifying R2R3-MYB proteins in other plants [16,18]. R2R3-MYB transcription factors are involved in regulating various biological processes in plants, such as growth and development, metabolic processes, and responses to environmental stresses. Additionally, the biological processes involving plant R2R3-MYB transcription factors show a clear correlation with the classification of R2R3-MYB proteins. In *Arabidopsis*, several R2R3-MYB transcription factors were involved in the regulation of flavonoid biosynthesis. The S7 group members, such as AtMYB11, AtMYB12, and AtMYB111, were involved in regulating flavonols biosynthesis [20]. The S6 group members, such as AtMYB75, AtMYB90, AtMYB113, and AtMYB114, have been confirmed to regulate anthocyanin biosynthesis [21]. AtMYB16, a transcription factor from the S9 group, was involved in the formation of petal epidermal cells, while AtMYB17 was involved in flower development and seed germination [31,32]. Similarly, NcMYB10, as a member of the S9 group, is speculated to play a role in flower development in *N. colorata*. Many R2R3-MYB transcription factors are also involved in plant responses to biotic and abiotic stresses in plants. AtMYB96, a transcription factor from the S1 group, regulated the responses to drought stress and disease resistance via the ABA signaling pathway [33,34]. AtMYB15 (S2 group) was involved in responses to cold stress [35], AtMYB2 (S20 group) regulated the expression of genes responsive to salt and drought stress [36], and AtMYB41 and AtMYB102 (S11 group) was involved in plant defense response [37,38,39]. It is speculated that members of the S2 and S20 groups in *N. colorata*, such as NcMYB23, NcMYB54, NcMYB62, and NcMYB78, may be involved in the response to abiotic stresses, while NcMYB6, NcMYB8, and NcMYB11 may be involved in responses to biotic stresses.

Several studies have shown that the expression of R2R3-MYB genes is regulated by various internal and external factors, with external factors primarily including biotic and abiotic stresses. In *Arabidopsis*, *AtMYB2* and *AtMYB96* genes were significantly upregulated in response to drought stress [33,36], and *AtMYB102* gene showed high responses to salt stress and wounding [39]. Many R2R3-MYB genes are regulated by miRNAs. For instance, miR159 targeted *AtMYB33*, *AtMYB35*, *AtMYB65*, and *AtMYB101* genes [40]; while miR858 targeted and silenced the expression of *AtMYB11*, *AtMYB13*, and *AtMYB20* genes [41]. Plant hormones are also intrinsic factors affecting the expression of R2R3-MYB genes. The *RhMYB108* gene was significantly upregulated after treatment with exogenous ethylene and JA [42]. The *TcMYB29a* gene from *Taxus chinensis* was strongly suppressed after treatment with exogenous methyl jasmonate (MeJA) [43]. The *CmMYB2* gene was upregulated after ABA treatment [44]. In this study, a *cis*-acting element analysis showed that all R2R3-MYB genes contained hormone response elements, with the highest number of ABA and MeJA response elements, suggesting that the expression of most R2R3-MYB genes in *N. colorata* may be regulated by ABA or MeJA signaling pathways.

Anthocyanin biosynthesis in plants is a part of the phenylpropanoid pathway [45]. The initial step in anthocyanin biosynthesis involves the CHS catalyzing the reaction between malonyl-CoA and coumaroyl-CoA to form naringenin chalcone. Naringenin chalcone is then converted to dihydromyricetin through the action of CHI, F3′H, and F3′5′H. DFR, a key enzyme in the anthocyanin biosynthetic pathway, catalyzes the conversion of dihydromyricetin into the colorless anthocyanin precursor leucodelphinidin, which is then converted to anthocyanins by ANS, marking the final step in anthocyanin synthesis [9,10]. Although UFGT does not directly participate in anthocyanin synthesis, it catalyzes the conversion of unstable anthocyanins into more soluble and stable anthocyanidins stored in cells [11]. Anthocyanin synthesis is primarily influenced by two types of genes: those involved in the biosynthetic pathway and regulatory genes, such as R2R3-MYB, bHLH, and WD40 transcription factors [46]. Many key genes in the anthocyanin biosynthesis pathway are regulated by R2R3-MYB transcription factors. The MdMYB3 transcription factor in apples activated the expression of *CHS* and *CHI* genes [25]. DFR and ANS are additional key enzymes in the anthocyanin biosynthesis pathway. The LvMYB1 transcription factor promoted anthocyanin synthesis by activating *LvANS* gene expression through binding to its promoter [26]. The PdMYB118 transcription factor from *Populus deltoids* regulated anthocyanin biosynthesis by binding to MYB-binding sites on the promoters of *DRF2* and *ANS1* genes [47]. In this study, we revealed the positive regulatory role of the NcMYB25 transcription factor in anthocyanin synthesis.

## 4. Materials and Methods

### 4.1. Identification, Properties, and Structural Analysis of R2R3-MYB in N. colorata

The genomic data of *N. colorata* were downloaded from the Genome Warehouse in BIG Data Center (accession number: GWHAAYW00000000) [6]. Genomic data for *Metasequoia glyptostroboides*, *Amborella trichopoda*, *Liriodendron chinense*, *Brasenia schreberi*, and *Euryale ferox* were downloaded from the NCBI database. MYB transcription factors were identified using HMMER v3.4 and the PF00249 model [48,49]. R2R3-MYB transcription factors were manually selected based on previously described sequence features of R2R3-MYB proteins [18]. The distribution of R2R3-MYB genes on chromosomes was visualized using Tbtools-II software [50]. Protein properties were predicted using the ProtParam tool from the Expasy database. The structure of R2R3-MYB proteins was analyzed using the MEME suite and visualized using Tbtools-II software [50].

### 4.2. Phylogenetic and Evolutionary Analysis

Multiple sequence alignments of R2R3-MYB proteins were performed using the MUSCLE algorithm [51], and the results were visualized using Jalview v2.11.4.0 software [52]. Phylogenetic trees were constructed using MEGA-X software [53]. R2R3-MYB transcription factors were classified based on the classification criteria of the R2R3-MYB factors in *Arabidopsis* [16,18]. Phylogenetic trees were visualized using the Evolview website [54]. Collinearity analysis was conducted using MCScanX software [55]. Ka, Ks, and Ka/Ks ratios were calculated using KaKs_Calculator 3.0 software [56]. Species trees and divergence times were obtained from the TimeTree website [57].

### 4.3. Expression Pattern Analysis

The region 2000 bp upstream of the gene start codon was used as a promoter sequence. *Cis*-acting elements in promoters were analyzed using the PlantCARE database [58]. To analyze expression patterns, 15 RNA-Seq datasets were downloaded from the Genome Sequence Archive (Accession Numbers: CRR058573~CRR058582 and CRR059175~CRR059179). The data from CRR058573~CRR058582 correspond to transcriptomic data from different tissues of *N. colorata* (mature leaf, mature leaf stalk, juvenile flower, juvenile leaf, juvenile leaf stalk, carpel, stamen, sepal, petal, and root), while the data from CRR059175~CRR059179 correspond to transcriptomic data from different tissues of the *White colorata* (stamen, sepal, petal, leaf, and carpel). Transcriptome data were analyzed on the Galaxy website, and gene expression levels were calculated using the featureCounts tool [59].

### 4.4. Total RNA Extraction and qRT-PCR Analysis

Fresh roots, stems, leaves, petioles, and petals of *N. colorata* were collected for RNA extraction. Total RNA was extracted using the TRIpure Total RNA Rapid Extraction Kit (Huayueyang Biotechnology (Beijing) Co., Ltd., Beijing, China). Reverse transcription was performed using the FastQuant RT Kit (With gDNase) (Tiangen Biotech (Beijing) Co., Ltd., Beijing, China) with 1 μg of total RNA. A 10-fold diluted cDNA solution was used as the template for qRT-PCR analysis. NcActin gene was used as the housekeeping gene in the qRT-PCR analysis. Primers for qRT-PCR were designed using Primer Premier 6 software; the primer sequences used for qRT-PCR are listed in Table S6. The qRT-PCR analysis was performed with FastFire qPCR PreMix (SYBR Green) (Tiangen Biotech (Beijing) Co., Ltd., Beijing, China) on QuantStudio 3 real-time PCR system (Thermo Fisher Scientific Inc., Waltham, MA, USA). The qRT-PCR reaction system (20 μL) included 10 μL PreMix, 2 μL of template, 0.6 μL each of forward and reverse primers, and 6.8 μL RNase-Free ddH_2_O. The qRT-PCR cycling conditions: initial denaturation at 95 °C for 1 min; 40 cycles of denaturation at 95 °C for 5 s, and annealing/extension at 60 °C for 15 s. Each experiment was conducted in triplicate. Relative gene expression levels were calculated using the 2^−ΔΔCt^ method [60].

### 4.5. Analysis of Possible Functions of R2R3-MYB Proteins in N. colorata

Functional analysis of R2R3-MYB proteins in *N. colorata* was performed using protein–protein interaction (PPI) and gene ontology (GO) functional analysis. PPI analysis was conducted using the STRING database [61]. GO functional annotation was performed using eggNOG-mapper v2 software [62].

### 4.6. Identification of miRNAs and Prediction of Target Genes of miRNAs

To identify miRNAs in *N. colorata*, a total of 7 ncRNA-Seq datasets were downloaded from the Genome Sequence Archive (SRX8123259, SRX8123358~SRX8123363). The miRDeep2 v2.0.1.2. software was used for miRNA identification [63]. The target genes of the miRNA were predicted using psRNATarget software (2017 Update) with default parameters [64].

### 4.7. Subcellular Localization Analysis Assay

Subcellular localization was analyzed using the tobacco transient transformation system [65,66]. The coding sequence (CDS) of the *NcMYB25* gene was cloned into the *pCAMBIA1305-GFP* vector to create the *pCAMBIA1305-NcMYB25-GFP* construct. This construct, along with the *pCAMBIA1305-GFP* vector, was transformed into *Agrobacterium tumefaciens* GV3101 separately. The *Agrobacterium* solution carrying the constructs was injected into the leaves of tobacco seedlings, and the plants were incubated under weak light for one day, then under normal conditions for 1–2 days. The fluorescence signal of GFP proteins and NcMYB25-GFP fusion proteins in tobacco leaves was observed using a laser scanning confocal microscope, with an excitation wavelength of 488 nm and an emission wavelength of 500–530 nm (Leica, Mannheim, Germany). Primers used for subcellular localization analysis are listed in Table S7.

### 4.8. Transcriptional Activation Analysis Assay

The transcriptional activation activity of the NcMYB25 transcription factor was analyzed using a yeast expression system [67]. The CDS of the *NcMYB25* gene was cloned into the *pGBKT7* vector to create the *pGBKT7-NcMYB25* construct. This construct and *pGBKT7* vector were transformed into *Saccharomyces cerevisiae* AH109 separately, and growth was tested on SD/–Trp and SD/–His/–Trp media. X-α-Gal (20 μg/mL) was added to SD/–His/–Trp media as an indicator of transcriptional activation activity. Primers used for transcriptional activation analysis assay are listed in Table S8.

### 4.9. Apple Pericarp Transient Transformation Assay

The apple pericarp transient transformation assay was performed following previously described methods [49]. The CDS of *NcMYB25* gene was cloned into the *pCAMBIA1303* vector to create the *pCAMBIA1303-NcMYB25* construct. This construct, along with the *pCAMBIA1303* vector, was transformed into *A. tumefaciens* GV3101 separately and injected into apple pericarps. The injected apples were placed in a dark incubator for 24 h, followed by 5 days in a lighted incubator. The apples inoculated with Agrobacterium containing the *pCAMBIA1303-NcMYB25* vector and those inoculated with Agrobacterium containing the *pCAMBIA1303* vector were maintained under identical environmental conditions. After this period, the color of the apple pericarp at the injection site was observed, and the anthocyanin concentration in the apple pericarp at the injection site was measured. The anthocyanin content in the pericarp was measured using the Anthocyanin Content Assay Kit (Biosharp Biotechnology Co., Ltd., Hefei, China). Primers used for apple pericarp transient transformation assay are listed in Table S9.

### 4.10. Statistical Analysis

A Student’s *t*-test was employed to determine the statistical significance of differences between two sample means, with “*” indicating significance at *p* < 0.05 and “**” indicating significance at *p* < 0.01. The statistical analysis was performed using Microsoft Office Excel 2019.

## 5. Conclusions

In this study, 59 R2R3-MYB genes were identified in *N. colorata*, distributed across 14 chromosomes. Segmental duplication and tandem duplication were the primary driving forces of the R2R3-MYB gene family formation in *N. colorata*. The R2R3-MYB transcription factors in *N. colorata* were likely involved in regulating flavonoid biosynthesis, plant growth and development, and responses to abiotic stress. We identified 92 miRNAs in *N. colorata*, with 43 interacting with 35 R2R3-MYB genes. The NcMYB25 transcription factor, which localized in the nucleus and possessed transcriptional activation activity, positively regulated anthocyanin synthesis. This study characterized the features of R2R3-MYB genes in *N. colorata* and the biological function of the *NcMYB25* gene in anthocyanin synthesis, contributing to the understanding of molecular mechanisms of flower color formation and providing data support for molecular breeding of water lily varieties.

## Data Availability

All data supporting this study are available within the paper and within the Supplementary Materials published online.

## Supplementary Materials

The following supporting information can be downloaded at: https://www.mdpi.com/article/10.3390/plants13212990/s1, Figure S1: Sequence conservation analysis of motifs in R2R3-MYB proteins of *N. colorata*; Figure S2: Multiple sequence alignment of R2R3-MYB proteins in *N. colorata*; Figure S3: Density curve of Ks in the *N. colorata* genome; Figure S4: PPI analysis of R2R3-MYB in *N. colorata*; Table S1: Physicochemical properties analysis of R2R3-MYB protein in *N. colorata*; Table S2: Analysis of evolutionary selective pressure of R2R3-MYB gene in *N. colorata*; Table S3: GO annotation of transcription factor R2R3-MYB in *N. colorata*; Table S4: The identification of miRNA in *N. colorata*; Table S5: Evolution history of R2R3-MYB gene in *N. colorata*; Table S6: Primers sequence for qRT-PCR assay; Table S7: Primers sequence for subcellular localization assay; Table S8: Primers sequence for transcription activation activity assay; Table S9: Primer sequences for transient expression assay in apple pericarps.
