# Supplementary material for: The Characterization of R2R3-MYB Genes in Water Lily Nymphaea colorata Reveals the Involvement of NcMYB25 in Regulating Anthocyanin Synthesis
Source: Plants (Basel). 2024 Oct 26;13(21):2990. doi: 10.3390/plants13212990 (PMC11548254; doi:10.3390/plants13212990)
Supplement: Supplementary file 1 [file plants-13-02990-s001.zip › Figure S2.pdf]

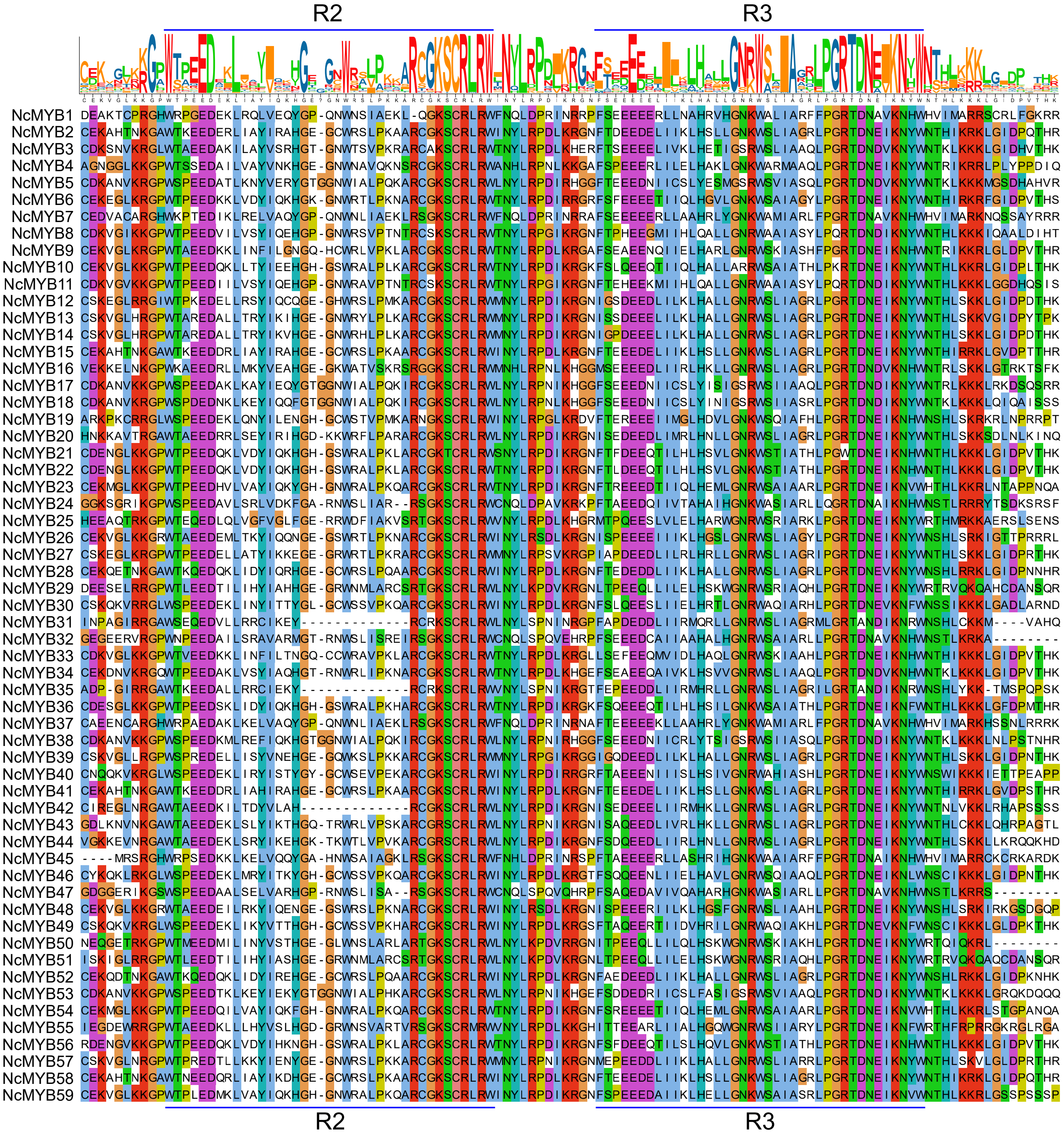
R2
R3
NcMYB1
NcMYB2
NcMYB3
NcMYB4
NcMYB5
NcMYB6
NcMYB7
NcMYB8
NcMYB9
NcMYB10
NcMYB11
NcMYB12
NcMYB13
NcMYB14
NcMYB15
NcMYB16
NcMYB17
NcMYB18
NcMYB19
NcMYB20
NcMYB21
NcMYB22
NcMYB23
NcMYB24
NcMYB25
NcMYB26
NcMYB27
NcMYB28
NcMYB29
NcMYB30
NcMYB31
NcMYB32
NcMYB33
NcMYB34
NcMYB35
NcMYB36
NcMYB37
NcMYB38
NcMYB39
NcMYB40
NcMYB41
NcMYB42
NcMYB43
NcMYB44
NcMYB45
NcMYB46
NcMYB47
NcMYB48
NcMYB49
NcMYB50
NcMYB51
NcMYB52
NcMYB53
NcMYB54
NcMYB55
NcMYB56
NcMYB57
NcMYB58
NcMYB59
R2
R3
