# Supplementary material for: The Characterization of R2R3-MYB Genes in Water Lily Nymphaea colorata Reveals the Involvement of NcMYB25 in Regulating Anthocyanin Synthesis
Source: Plants (Basel). 2024 Oct 26;13(21):2990. doi: 10.3390/plants13212990 (PMC11548254; doi:10.3390/plants13212990)
Supplement: Supplementary file 1 [file plants-13-02990-s001.zip › Supplementary_Figure_legends.pdf]

Supplementary Figure Legends

Figure S1. Sequence conservation analysis of motifs in R2R3-MYB proteins of *N. colorata*. Blue boxes indicated the components of the R2 conserved domain, while red boxes represented the components of the R3 conserved domain.

Figure S2. Multiple sequence alignment of R2R3-MYB proteins in *N. colorata*. The sequence shown was the trimmed result of the multiple sequence alignment. Visualization was done using the 'Clustal' color scheme in Jalview software.

Figure S3. Density curve of Ks in the *N. colorata* genome

Figure S4. PPI analysis of R2R3-MYB in *N. colorata*.
