# Supplementary material for: The Characterization of R2R3-MYB Genes in Water Lily Nymphaea colorata Reveals the Involvement of NcMYB25 in Regulating Anthocyanin Synthesis
Source: Plants (Basel). 2024 Oct 26;13(21):2990. doi: 10.3390/plants13212990 (PMC11548254; doi:10.3390/plants13212990)
Supplement: Supplementary file 1 [file plants-13-02990-s001.zip › Table.pdf]

Supplementary tables:

Table S1 Physicochemical properties analysis of R2R3-MYB protein in *N. colorata*

Gene ID	Gene Name	Number of Amino Acid	Molecular Weight/kD	Theoretical pI	Instability Index	GRAVY
NC1G0091020	NcMYB1	319	35.71	7.11	54	–0.661
NC1G0192860	NcMYB2	264	29.39	7.54	57.35	–0.541
NC1G0193200	NcMYB3	389	43.46	4.98	46.79	–0.58
NC1G0134540	NcMYB4	513	55.06	6.05	60.54	–0.425
NC1G0131980	NcMYB5	319	34.7	7.07	57.82	–0.503
NC1G0136170	NcMYB6	336	37.71	6	47.88	–0.532
NC1G0065480	NcMYB7	447	49.19	6.86	52.45	–0.565
NC1G0065230	NcMYB8	304	34.15	8.32	56.6	–0.643
NC2G0004270	NcMYB9	262	29.42	5.6	43.23	–0.644
NC2G0033460	NcMYB10	442	48.5	8.57	58.78	–0.623
NC2G0033860	NcMYB11	366	40.18	6.52	55.63	–0.67
NC2G0035230	NcMYB12	313	35.35	8.62	57.34	–0.742
NC2G0035240	NcMYB13	298	33	7.14	48.78	–0.559
NC2G0035260	NcMYB14	304	33.67	8.88	42.5	–0.599
NC2G0037380	NcMYB15	281	31.51	9.15	57.18	–0.517
NC3G0229380	NcMYB16	287	32.89	5.8	45.76	–0.981
NC3G0225940	NcMYB17	336	37.51	5.35	55.42	–0.616
NC3G0228100	NcMYB18	335	37.47	6.53	52.34	–0.618
NC4G0019470	NcMYB19	375	41.96	5.84	66.91	–0.465
NC4G0021250	NcMYB20	273	31.15	6.12	46.47	–0.794
NC4G0022430	NcMYB21	142	16.39	9.41	34.46	–0.766
NC4G0022610	NcMYB22	322	36.16	5.98	45.19	–0.668
NC4G0237140	NcMYB23	297	33.41	5.19	54.67	–0.607
NC5G0048790	NcMYB24	420	46.07	8.99	47.38	–0.609
NC5G0263650	NcMYB25	281	31.54	6.27	62.87	–0.539
NC5G0161710	NcMYB26	423	47.59	4.68	57.81	–0.822
NC6G0024470	NcMYB27	349	38.48	6.78	61.63	–0.818
NC7G0247160	NcMYB28	254	28.17	9.14	49.6	–0.657
NC7G0176470	NcMYB29	292	33.77	7.12	63.14	–0.749
NC7G0176450	NcMYB30	320	35.84	6.31	65.12	–0.515
NC8G0213380	NcMYB31	288	32.75	6.13	61.21	–0.658
NC8G0216870	NcMYB32	229	24.5	6.91	59.32	–0.566
NC8G0216500	NcMYB33	341	38.14	5.15	50.87	–0.46
NC8G0215960	NcMYB34	379	41.24	6.79	40.58	–0.491
NC8G0213420	NcMYB35	257	29.01	6.16	66	–0.634
NC8G0218300	NcMYB36	334	37.2	5.94	48.68	–0.637

Gene ID	Gene Name	Number of Amino Acid	Molecular Weight/kD	Theoretical pI	Instability Index	GRAVY
NC9G0234200	NcMYB37	449	49.16	6.62	50.21	–0.628
NC9G0170240	NcMYB38	336	37.76	7.06	53.44	–0.66
NC9G0273470	NcMYB39	358	40.31	9.33	44.28	–0.493
NC9G0275540	NcMYB40	312	34.45	5.67	56.59	–0.499
NC9G0276120	NcMYB41	225	25.46	9.09	49.86	–0.694
NC10G0031990	NcMYB42	286	31.67	5.95	63.06	–0.81
NC10G0032020	NcMYB43	274	31.11	7.04	44.59	–0.835
NC10G0032030	NcMYB44	239	27.83	9.07	44.8	–0.903
NC10G0248370	NcMYB45	233	26.85	9.77	64.7	–0.864
NC10G0166400	NcMYB46	435	48.24	5.89	60.07	–0.626
NC10G0167650	NcMYB47	237	25.77	8.38	67.9	–0.583
NC10G0045550	NcMYB48	458	51.43	8.57	56.61	–0.655
NC11G0120250	NcMYB49	345	39.11	7.68	55.72	–0.701
NC11G0124080	NcMYB50	218	24.46	5.76	46	–0.882
NC11G0244500	NcMYB51	311	35.29	6.53	54.68	–0.601
NC11G0302390	NcMYB52	303	34.04	9.45	68.25	–0.734
NC12G0096940	NcMYB53	333	37.24	7.03	43.68	–0.613
NC12G0249440	NcMYB54	320	35.65	5.41	54.13	–0.509
NC12G0188880	NcMYB55	264	31.22	9.09	65.25	–0.895
NC12G0185110	NcMYB56	317	35.28	6.06	51.8	–0.537
NC13G0025670	NcMYB57	342	38.35	9.57	56.32	–0.74
NC13G0058960	NcMYB58	275	30.98	8.46	45.98	–0.641
NC14G0174460	NcMYB59	341	38.7	5.28	62.63	–0.798

Note: GRAVY is short for the grand average of hydropathicity.

Table S2 Analysis of evolutionary selective pressure of R2R3-MYB gene in *N. colorata*

Gene pair	Ka	Ks	Ka/Ks	Selection Pressure	Duplication type
NcMYB8-NcMYB11	0.321	NaN	NaN	Purifying selection	Segmental duplication
NcMYB12-NcMYB39	0.420	1.353	0.311	Purifying selection	Segmental duplication
NcMYB15-NcMYB41	0.097	1.265	0.077	Purifying selection	Segmental duplication
NcMYB18-NcMYB38	0.289	1.561	0.185	Purifying selection	Segmental duplication
NcMYB22-NcMYB56	0.192	1.218	0.157	Purifying selection	Segmental duplication
NcMYB23-NcMYB54	0.138	0.819	0.168	Purifying selection	Segmental duplication
NcMYB26-NcMYB48	0.275	2.106	0.131	Purifying selection	Segmental duplication
NcMYB13-NcMYB14	0.133	0.388	0.342	Purifying selection	Tandem duplication
NcMYB29-NcMYB30	0.685	2.311	0.296	Purifying selection	Tandem duplication
NcMYB43-NcMYB44	0.361	2.956	0.122	Purifying selection	Tandem duplication

Table S3 GO annotation of transcription factor R2R3-MYB in *N. colorata*

The contents of Table S3 were stored in a file named **Table S8.xlsx**.

Table S4 The identification of miRNA in *N. colorata*

The contents of Table S4 were stored in a file named **Table S9.xlsx**.

Table S5 Evolution history of R2R3-MYB gene in *N. colorata*

+4	+27/-1		+12	+9/-4		+2/-5		+8
	+27	-1		+9	-4	+2	-5	
NcMYB11	*NcMYB5*	*NcMYB39*	*NcMYB4*	*NcMYB2*	*NcMYB6*	*NcMYB44*	*NcMYB7*	*NcMYB1*
NcMYB12	*NcMYB6*		*NcMYB9*	*NcMYB3*	*NcMYB10*	*NcMYB57*	*NcMYB35*	*NcMYB13*
NcMYB36	*NcMYB7*		*NcMYB11*	*NcMYB16*	*NcMYB27*		*NcMYB43*	*NcMYB14*
NcMYB39	*NcMYB8*		*NcMYB12*	*NcMYB33*	*NcMYB55*		*NcMYB55*	*NcMYB21*
	NcMYB10		*NcMYB19*	*NcMYB35*			*NcMYB58*	*NcMYB29*
	NcMYB15		*NcMYB22*	*NcMYB45*				*NcMYB30*
	NcMYB17		*NcMYB23*	*NcMYB49*				*NcMYB31*
	NcMYB18		*NcMYB32*	*NcMYB52*				*NcMYB37*
	NcMYB20		*NcMYB36*	*NcMYB58*				
	NcMYB24		*NcMYB43*					
	NcMYB25		*NcMYB47*					
	NcMYB26		*NcMYB56*					
	NcMYB27							
	NcMYB28							
	NcMYB34							
	NcMYB38							
	NcMYB40							
	NcMYB41							
	NcMYB42							
	NcMYB46							
	NcMYB48							
	NcMYB50							
	NcMYB51							
	NcMYB53							
	NcMYB54							
	NcMYB55							
	NcMYB59							

Table S6 Primers sequence for qRT-PCR assay

Primer name	Primer sequence
NcMYB2-S	TGCCAGGAAGGACGGACAATGA
NcMYB2-AS	GGATCAATGCCTCGGCTCAACA
NcMYB10-S	CATGGTCAGGTGGTCGGCAATC
NcMYB10-AS	ATCGGACTTGGGCTTGTGGGT
NcMYB11-S	GCAGCCATTGCTTCCTACCTCC
NcMYB11-AS	TCGCCACCACCACCGCTAAT
NcMYB13-S	CAGACCAAGCGTCCTACCACCA
NcMYB13-AS	AGCCACCACCATCCACCGAAT
NcMYB23-S	AACCAGGCGACCAGCACCAT
NcMYB23-AS	GCCGTCGTAGGGACACAGGATT
NcMYB24-S	GATGGTGCTGCTGATGGCTCTC
NcMYB24-AS	TGACGGACACTCCCTTCCAGAT
NcMYB26-S	GGAGGTAGAGAAGCCGTTCAGAGT
NcMYB26-AS	TCACAGCAGCAAGAATCCATCACC
NcMYB27-S	ACCTCCACCTCCTCCTCCTTGT
NcMYB27-AS	CAGCATCATCATCTCGCCTCGG
NcMYB28-S	CGGACCTGAACCTGGAATTGACC
NcMYB28-AS	GCCGAAGAGGAGACATGGAGGAT
NcMYB35-S	GGAGGAGTTGGAAGAGAAGGAGGA
NcMYB35-AS	GCCGCTATAGGAGTTGTTGCTTGA
NcMYB41-S	CCGCCTCCGCTGGATCAATTATC
NcMYB41-AS	GTCCTTCCTGGTAGTCTTCCTGCT
NcMYB43-S	CACACCACTACTGCCGCAACTG
NcMYB43-AS	GTCGCTGCCACCATCACAATCT
NcMYB46-S	ATGGTTCCTCCCGTCACTCTGA
NcMYB46-AS	TGCTGCCGCTACTGCTGCTA
NcMYB48-S	GGAGGCATGACAAAGGCGGAAG
NcMYB48-AS	GGCTGTTGGATGTGGAGGAAGG
NcMYB50-S	TGGAGGCGGCAATTCTGGTGAT
NcMYB50-AS	CCCATGTGTTGTCGCTGTGTTCA
NcMYB52-S	GGACGAAGCAGGAGGACCAGAA
NcMYB52-AS	CTCTTCCCACACCGCAGCAATC
NcMYB53-S	CCAACAGCAGGAGCAGCAGAAT
NcMYB53-AS	TGGTGGTGGTCGAGGTTGAGTG
NcMYB54-S	CCTGCTGCGAGAAGATGGGACT
NcMYB54-AS	GCCCTCCAATTTCCGTGACCAAA
NcMYB58-S	AGGAAGGCTGCCAGGAAGAACA
NcMYB58-AS	CCTGAGAGTGCGGTTGGTTGAG

Primer name	Primer sequence
NcMYB59-S	CGCAGCTATCGTTCTTCGACCTT
NcMYB59-AS	ACATTCACCGCCTCCTCATCCA
NcActin-S	CCGATGTCCAGAAGTGCTGTTCC
NcActin-AS	GGCAGTGATCTCCTTGCTCATACG

Table S7 Primers sequence for subcellular localization assay

Primer name	Primer sequence
1305-NcMYB25-F	agtccggagctagctctagaATGGAAGCTCTATTAATACGAG
1305-NcMYB25-R	cccttgctcaccatggatccGAGAAAATGGTCCCCAACAGAG

Table S8 Primers sequence for transcription activation activity assay

Primer name	Primer sequence
pGBKT7-NcMYB25-F	tggccatggaggccgaattcATGGAAGCTCTATTAATACGAG
pGBKT7-NcMYB25-R	cgctgcaggtcgacggatccGAGAAAATGGTCCCCAACAGAG

Table S9 Primer sequences for transient expression assay in apple peel

Primer name	Primer sequence
1303-NcMYB25-F	atgaccatgattacgaattcATGGAAGCTCTATTAATACGAG
1303-NcMYB25-R	cgacggccagtgccaagcttGAGAAAATGGTCCCCAACAGAG
