# Supplementary figures and images for: The Characterization of R2R3-MYB Genes in Water Lily Nymphaea colorata Reveals the Involvement of NcMYB25 in Regulating Anthocyanin Synthesis
Source: Plants (Basel). 2024 Oct 26;13(21):2990. doi: 10.3390/plants13212990 (PMC11548254; doi:10.3390/plants13212990)

R2 R3

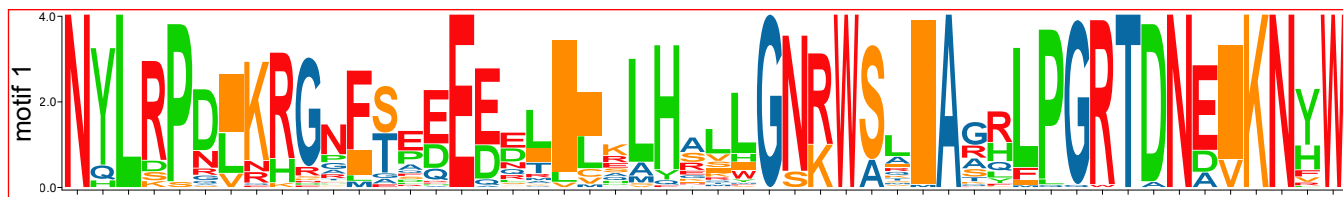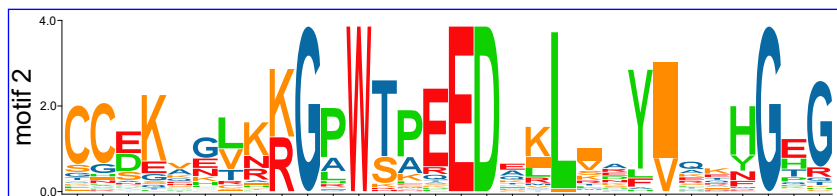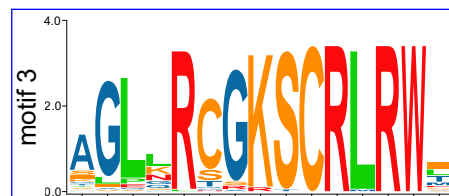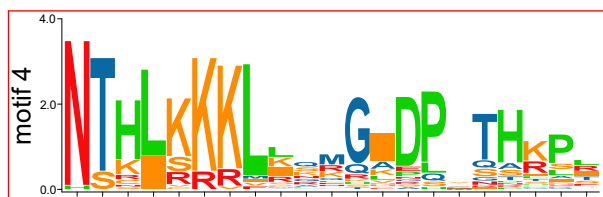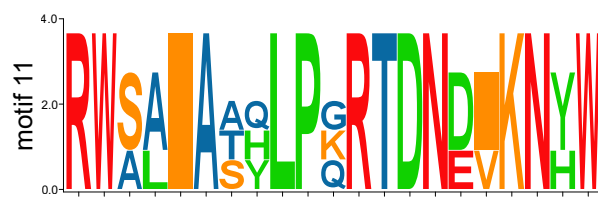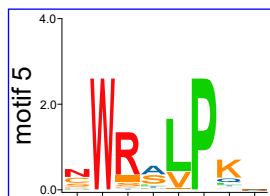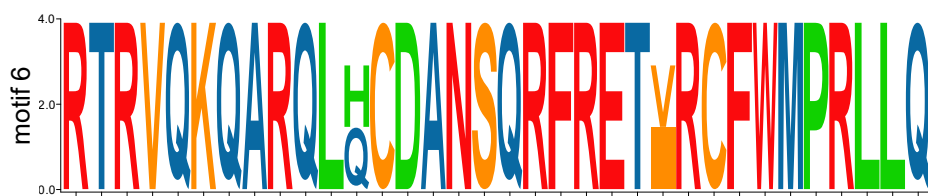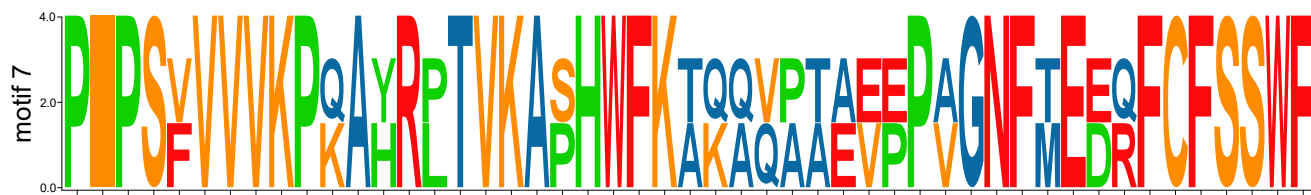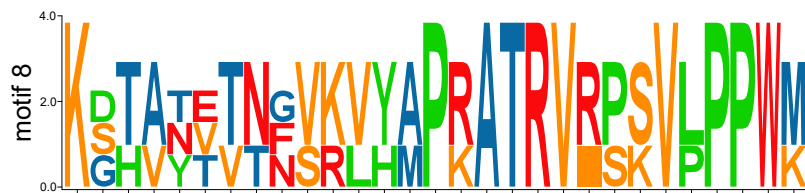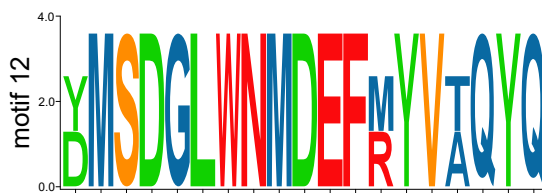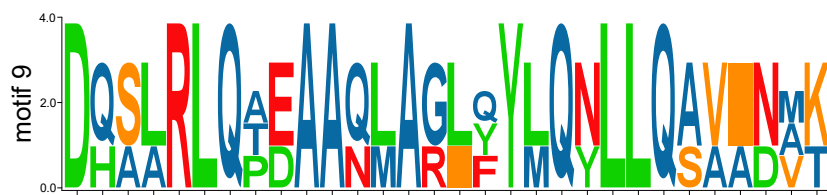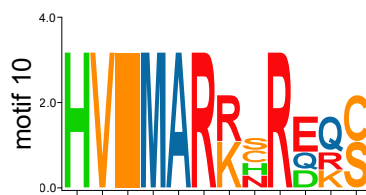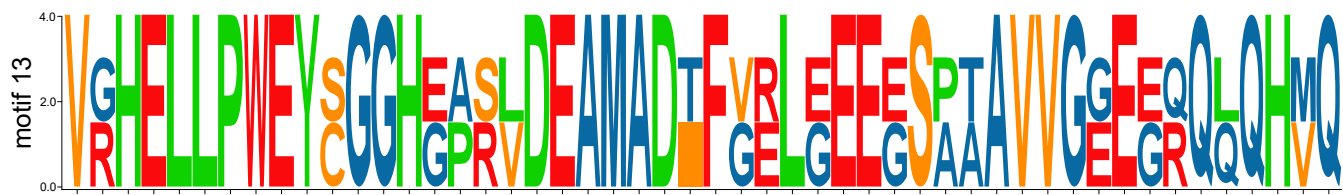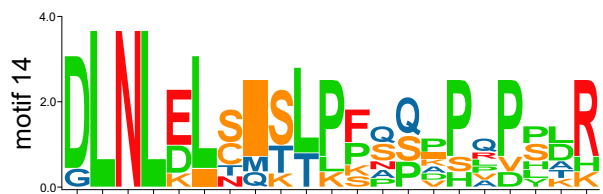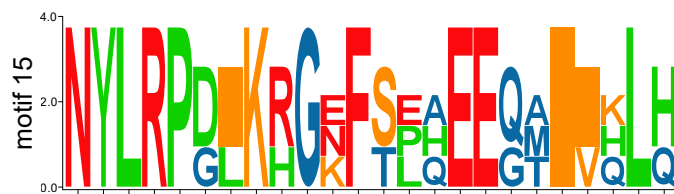

Supplement: Supplementary file 1 [file plants-13-02990-s001.zip › Figure S1.pdf]

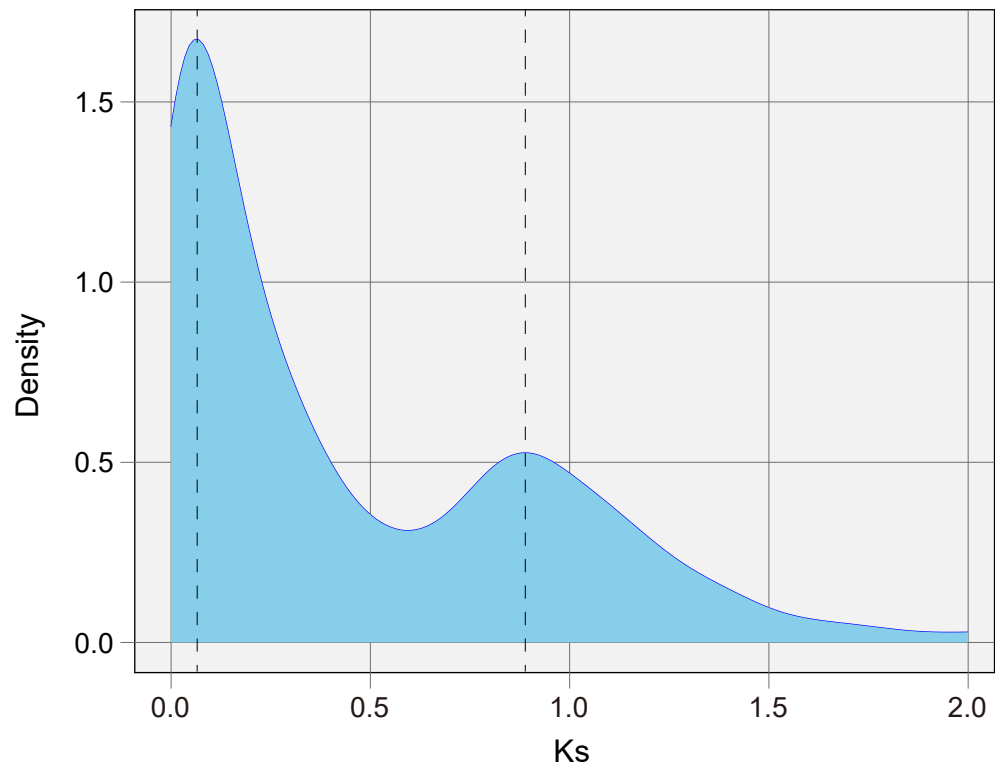

Supplement: Supplementary file 1 [file plants-13-02990-s001.zip › Figure S3.pdf]

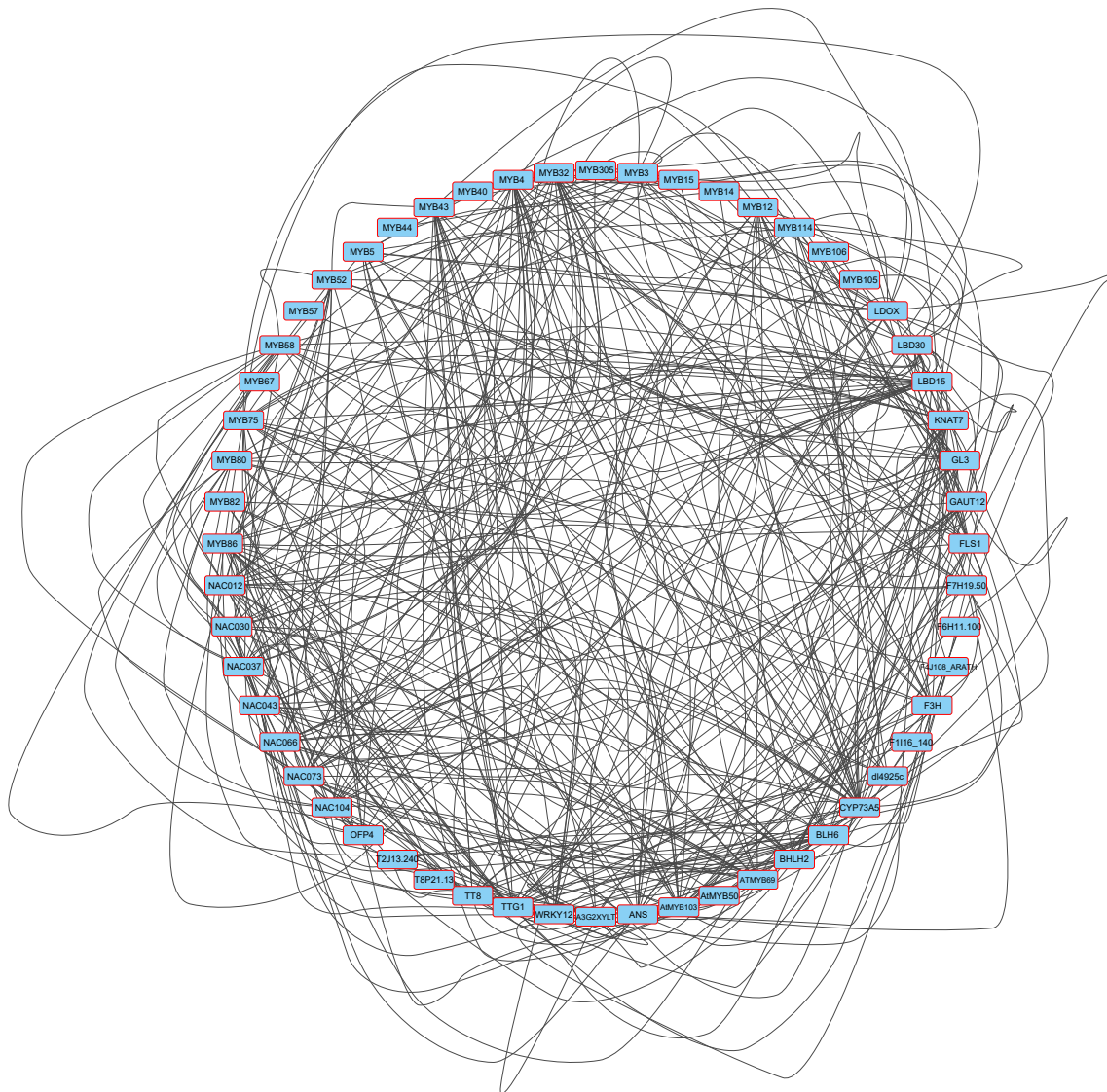

Supplement: Supplementary file 1 [file plants-13-02990-s001.zip › Figure S4.pdf]
